# Polyamorphism in Yb-based metallic glass induced by pressure

**DOI:** 10.1038/srep46762

**Published:** 2017-04-25

**Authors:** Liangliang Li, Qiang Luo, Renfeng Li, Haiyan Zhao, Karena W. Chapman, Peter J. Chupas, Luhong Wang, Haozhe Liu

**Affiliations:** 1 Harbin Institute of Technology, Harbin 150080, China; 2 Center for High Pressure Science and Technology Advanced Research, Changchun 130015, China; 3 School of Materials Science and Engineering, Tongji University, Shanghai 201804, China; 4 Center for Advanced Energy Studies, University of Idaho, Idaho Falls, Idaho 83406, USA; 5 X-ray Science Division, Advanced Photon Source, Argonne National Laboratory, Argonne, Illinois 60439, USA

## Abstract

The Yb_62.5_Zn_15_Mg_17.5_Cu_5_ metallic glass is investigated using synchrotron x-ray total scattering method up to 38.4 GPa. The polyamorphic transformation from low density to high density with a transition region between 14.1 and 25.2 GPa is observed, accompanying with a volume collapse reflected by a discontinuousness of isothermal bulk modulus. This collapse is caused by that distortional icosahedron short range order precedes to perfect icosahedron, which might link to Yb 4*f* electron delocalization upon compression, and match the result of *in situ* electrical resistance measurement under high pressure conditions. This discovery in Yb-based metallic glass, combined with the previous reports on other metallic glass systems, demonstrates that pressure induced polyamorphism is the general behavior for typical lanthanide based metallic glasses.

Traditional network-forming glassy state materials^1^, such as ice^2^, silica^3^ and silicon^4^ have been reported to exhibit polyamorphism induced by pressure, which tends to be ascribed to an increase in atomic coordination. Whereas, metallic glasses are distinct from the traditional glasses due to the non-directional metallic bonds. Therefore, polyamorphism induced by pressure in metallic glass was supposed to be impossible for they are spatially densely-packing and have the maximum coordination number already. However, polyamorphic transitions were discovered in Ce-based metallic glasses attributed to 4*f* electron delocalization in Ce under high pressure^5^,^6^. This rises a question that it is the general behavior for all lanthanide based metallic glasses, or not.

Ytterbium, as an end member of the rare-earth lanthanides, behaves irregular in comparison with other members of the lanthanide series owing to its special electronic configuration. At ambient conditions, ytterbium is considered as divalent with an electronic configuration close to 4*f* ^14^(5*d*6*s*)^2^, while the other lanthanides as trivalent state 4*f*^ *n*^(5*d*6*s*)^3^, where *n* varies from 0 to 14 corresponding to lanthanum to lutetium. The special electronic configuration of ytterbium results in a special sequence of phase transitions at high temperatures as well as high pressures^7^,^8^,^9^,^10^. If Ce with electronic configuration of 4*f*^*1*^(5*d*6*s*)^3^ could be looked as “*n*-type one electron” like beginning member, Yb, on the other hand, could be looked as 4*f* ^14^(5*d*6*s*)^3+(−1)^, i. e. “*p*-type one hole like” ending member, which indeed results in a *d*- rather than an *f* differentiating electron behavior. Therefore, searching the potential polyamorphic transformation in Yb-based metallic glasses under compression conditions will be interesting to reveal whether the generality of polyamorphism exists.

Generally, pressure-volume studies are particularly valuable in confirming whether polyamorphic transitions occur^10^. Such polyamorphic transformations derived from valence transitions should involve in changes of electrical transport property. In this work, to shed some light on the nature of polyamorphic transitions induced by pressure in Yb-based metallic glass systems, a study was performed by means of synchrotron x-ray total scattering and resistance measurements, focusing on another side of ending member of this lanthanide base metallic glass family, namely Yb_62.5_Zn_15_Mg_17.5_Cu_5_ metallic glass.

## Results and Discussion

### X-ray total scattering under pressure

**Figure 1:**
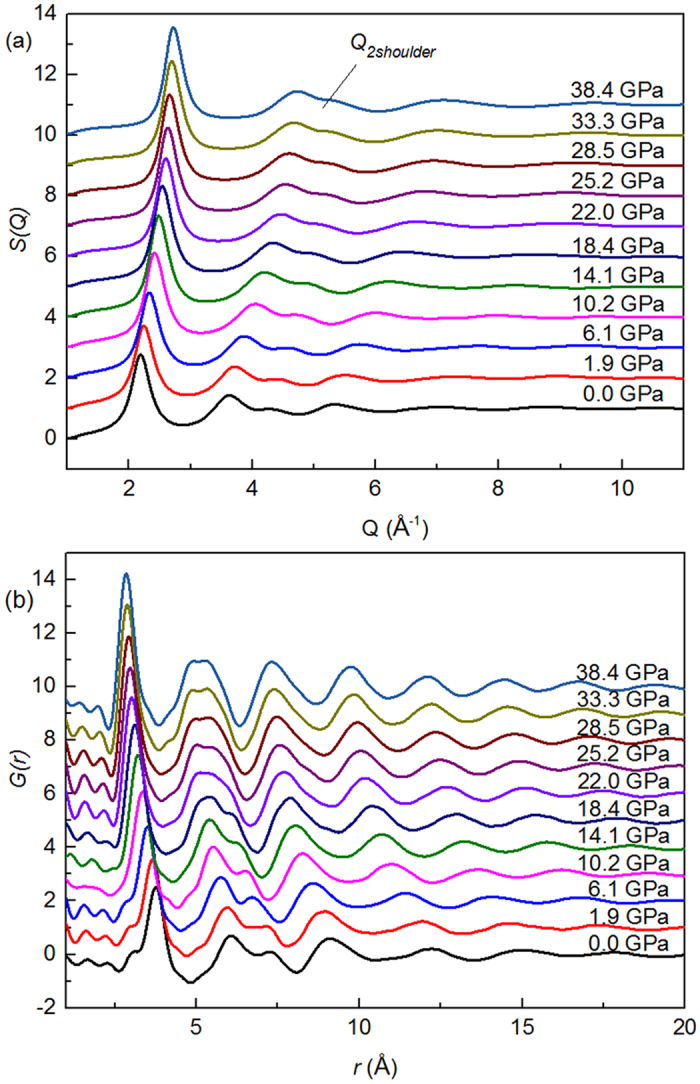
(**a**) Structure factor *S(Q)* and (**b**) pair distribution function *G(r)* of the Yb_62.5_Zn_15_Mg_17.5_Cu_5_ metallic glass at various pressure conditions.

**Figure 2:**
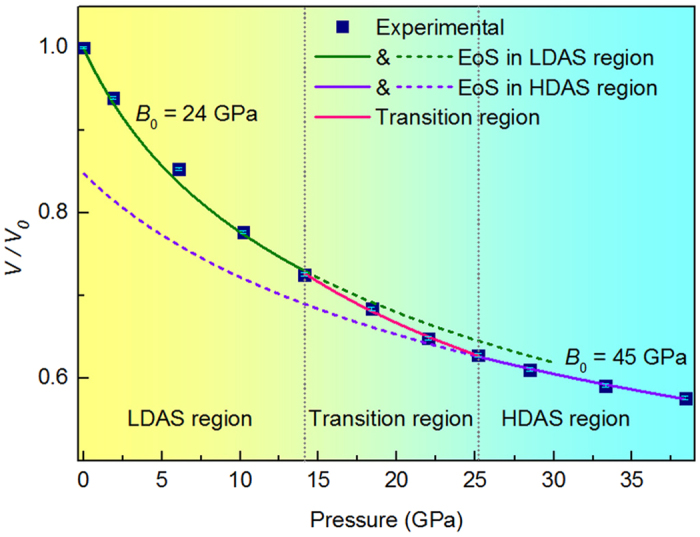
Relative volume *V/V*_*0*_ of Yb_62.5_Zn_15_Mg_17.5_Cu_5_ metallic glass as a function of pressure, where *V*_*0*_ is initial volume. The green and purple line show the EoS fitting results at LDAS region and HDAS region, respectively. The pink line is for transition region.

The structure factor *S(Q)* and corresponding PDFs of the Yb_62.5_Zn_15_Mg_17.5_Cu_5_ metallic glass extracting from synchrotron x-ray scattering experiment as a function of pressure are displayed in Fig. 1. Absence of sharp peaks in *S(Q)* and PDFs indicates that the sample remains fully amorphous state in the entire pressure range. As expected for the compression effect, first peak position *Q*_*1*_ of structure factor *S(Q)* in reciprocal space shifts towards higher momentum transfer and the nearest-neighbor distance *r*_*1*_ in real space shifts to shorter distance with increasing pressure. The shifts of *Q*_*1*_ of structure factor *S(Q)* and the nearest-neighbor distance *r*_*1*_ reflect changes in volume or density induced by pressure, which is illustrated by a power law^3^,^11^,^12^,^13^. Using normalized first peak position *Q*_*1*_ with a power of 2.5 to estimate the relative volume change as a function of pressure, a fast decrease of the average atomic volume under pressure range from 14.1 to 25.2 GPa, a transition region, is observed. Two amorphous states, low density amorphous states (LDAS) and high density amorphous states (HDAS), were separated by the transition region. Fitting the pressure-volume data by the second-order Birch-Murnaghan equation of state (EoS)^14^, the isothermal bulk modulus are determined as *B*_*0*_ = 24(1) GPa and 45(7) GPa at different state region respectively, presenting a discontinuousness of isothermal bulk modulus, as shown in Fig. 2. The volume difference between the two states is as large as 15.2% at ambient pressure. Furthermore, the volume collapse taking place continuously and undergoing a broad pressure range rather than abruptly at certain pressure point shows an anomalous compressed region, which is in agreement with polyamorphic transition in Ce_55_Al_45_ metallic glass with a smooth and continuous transition region between 2 and 13.5 GPa^5^. This anomalous compressed curve is also observed in phase transition of ytterbium monotelluride under pressure^15^.

**Figure 3:**
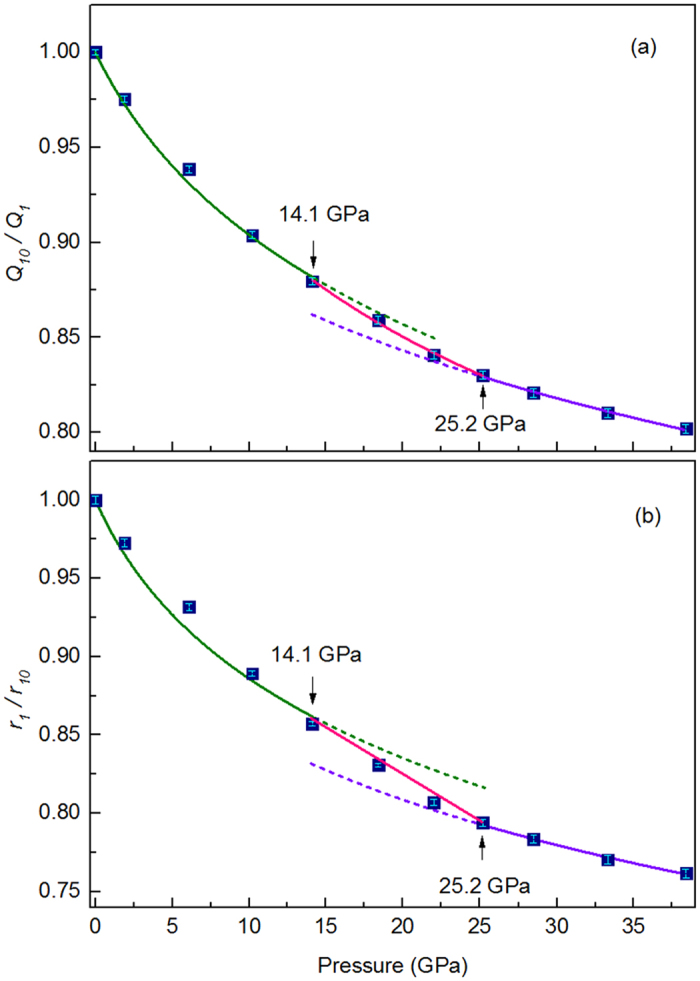
Pressure dependence of (**a**) normalized first peak position *Q*_*1*_ and (**b**) normalized nearest-neighbor distance *r*_*1*_, respectively, where *Q*_*10*_ and *r*_*10*_ are initial first peak position and nearest-neighbor distance, respectively. Equation used in fitting both *Q*_*10*_/*Q*_*1*_ and *r*_*1*_*/r*_*10*_ as a function of pressure is based on second-order Birch-Murnaghan EoS.

As shown in Fig. 3(a,b), both *Q*_*1*_ and *r*_*1*_ move with a relatively fast pace at transition region and the changes of both *Q*_*1*_ and *r*_*1*_as a function of pressure exhibit a trend similar to that of volume, which suggest that the polyamorphic transformation is embodied as well in medium range and short range length scale besides long range length scale. Intuitive local structure change of Yb_62.5_Zn_15_Mg_17.5_Cu_5_ within short range length scale induced by pressure was reflected by gradually disappeared the low-r shoulder of the first peak in G(*r*), as shown in Fig. 1(b).

**Figure 4:**
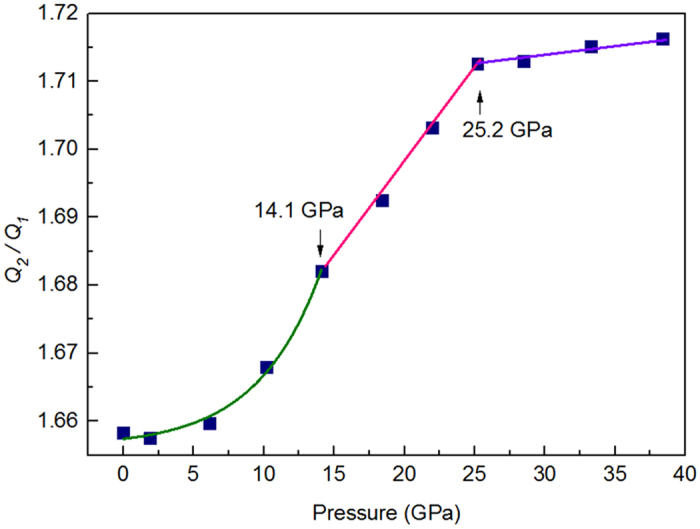
*Q*_*2*_*/Q*_*1*_ in the Yb_62.5_Zn_15_Mg_17.5_Cu_5_ metallic glass as a function of pressure.

As is displayed in Fig. 1, the second peak in *S (Q)* and PDFs are of splitting at each pressure point which are characteristic for conventional amorphous alloy systems^16^,^17^. In real space, the changes splitting of second peak under pressure reflect structural changes of Yb_62.5_Zn_15_Mg_17.5_Cu_5_ in medium range length scale. Such change is consistent with phase transition characterized by shifts of *Q*_*1*_which embody the medium range order. In reciprocal space the relationship between the first peak and splitting second peak as a function of pressure is indicative of short range order change^18^,^19^. Define position of the second peak and its shoulder as *Q*_*2*_ and *Q*_*2shoulder*_. Using two Gaussian functions to fit the second peak, the ratios of peak position show *Q*_*2shoulder*_/*Q*_*1*_ = 1.93 and *Q*_*2*_*/Q*_*1*_ varying from 1.66 to 1.72 with increased pressure, as displayed in Fig. 4. In contrast to a perfect icosahedron short range order, *Q*_*2shoulder*_/*Q*_*1*_ = 2.04 and *Q*_*2*_/*Q*_*1*_ = 1.71^20^, short range order changes in the Yb_62.5_Zn_15_Mg_17.5_Cu_5_ metallic glass under pressure go through from a more distortional icosahedra to a more perfect one with a different pace corresponding to different phase states. The polyamorphic transformation in the Yb_62.5_Zn_15_Mg_17.5_Cu_5_ metallic glass under pressure is thus mainly related to the reduction of icosahedra distortion with a different pace leading to volume collapse. This result is consistent with the change of PDFs whose each peak is sharper increasingly with increasing pressure, indicating the structure gradually becomes more order compared to initial structure, as presented in Fig. 1(b). At stage of LDAS, the change of distortion with pressure is slight and it contributes to a weak volume reduction. The diminution in degree of icosahedra distortion proceeds with faster pace within a transition region, then, distortional icosahedron is close to a more perfect one reaching the range of HDAS, which leads to a large volume change. Whereas, such changes of distortional icosahedra result from the decrease of average atomic distance in first neighbor shell with a different step as shown in Fig. 3(b), which should be radically associated with electronic transformation.

The phase transition of the Yb_62.5_Zn_15_Mg_17.5_Cu_5_ metallic glass can be ascribed to the electronic transition. In the Yb_62.5_Zn_15_Mg_17.5_Cu_5_ metallic glass, the 4*f* shell of Yb is completely full and considered as divalent with an electronic configuration close to 4*f*^14^(5*d*6*s*)^2^ at ambient conditions, whereas, under high pressure conditions, it transforms to a trivalent state 4*f* ^13^(5*d*6*s*)^3^ when one of the localized 4*f* electrons is delocalized, which is deduced from mechanism lied in polymorphism in ytterbium compounds^9^,^21^ and polyamorphism in Ce-based metallic glass under pressure^5^,^6^. An Yb in the trivalent state has a smaller atomic volume compared to divalent state, and this results in a striking decrease in average bond length, which coincides with the change of icosahedral distortion and volume collapse.

### The pressure dependence of electrical resistivity

**Figure 5:**
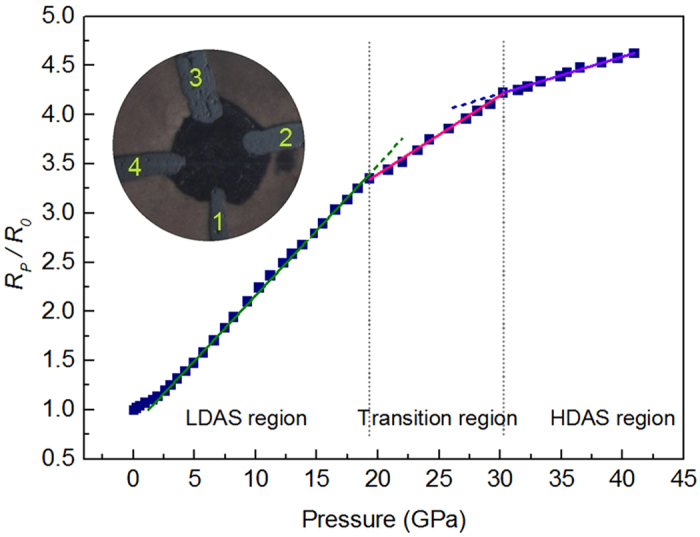
Relative resistance of the Yb_62.5_Zn_15_Mg_17.5_Cu_5_ metallic glass as a function of pressure. The inset shows the microphotograph of the sample with four Pt electrodes in chamber between two anvils at about 5 GPa conditions.

Electronic transition in the Yb_62.5_Zn_15_Mg_17.5_Cu_5_ metallic glass should be reflected in changes of electronic transport property. Therefore, an *in situ* resistance measurement under compression conditions was carried out. Figure 5 displays the resistance of Yb_62.5_Zn_15_Mg_17.5_Cu_5_ metallic glass as a function of pressure. According to weak-scattering theory which is well suited for low-resistivity metallic glasses, the resistance of a metallic glass is considered as arising from disordered atom scattering described by structure factor^22^,^23^. The pressure dependence of electrical resistivity is effected by several factors, such as the Fermi energy, the electron-ion interaction and structure factor of the system. As a result of a rather delicate interplay of these factors, the resistivity of metallic glasses is either to increase or to decrease under compressed conditions depending on different systems^24^,^25^. In the Yb_62.5_Zn_15_Mg_17.5_Cu_5_ metallic glass system, a positive pressure coefficient of the resistivity (PCR) is observed. Additionally, both the structure and electronic transition are arising from Yb 4*f* electrons delocalization as well as relation between pressure and electrical resistivity is volume dependence^23^, therefore, resistance of the Yb_62.5_Zn_15_Mg_17.5_Cu_5_ metallic glass exhibits a similar transition region, which is from 19.3 to 30.2 GPa, to that of low density to high density transition displayed in synchrotron x-ray scattering experiment. The shift of transition region might be due to quasi-hydrostatic pressure conditions in resistance measurement experiment. The pressure effect on this metallic glass is basically reversible, but with sluggish effect when pressure released. Thus this polyamorphism could only be studied *via in situ* high pressure methods.

The positive PCR obviously reduced after transition in the Yb_62.5_Zn_15_Mg_17.5_Cu_5_ metallic glass is observed in Fig. 5. Cheung and Ashcroft^26^ found a positive PCR for the polyvalent metals Mg, Al, Sn and a negative PCR for the monovalent metal K in liquid metals under pressure. Particularly, the positive PCR is maximal for the divalent metals^22^,^25^. This conclusion may suit for Yb contribution to PCR of the Yb_62.5_Zn_15_Mg_17.5_Cu_5_ metallic glass as well. The conversion from divalent to trivalent Yb leads to that contribution of Yb to positive PCR in the Yb_62.5_Zn_15_Mg_17.5_Cu_5_ metallic glass decreases in case of 4*f* electron delocalization, which gives rise to decline of positive PCR after transition.

A polyamorphic transformation from low density to high density with a transition region between 14.1 and 25.2 GPa occurs accompanying with change of electrical transport property. The transitions could be explained by 4*f* electron delocalization in Yb. The 4*f*–5*d* electronic collapse is reflected in both pressure-volume and pressure-resistivity relationship. This findings, combined with the previous reports on pressure induced polyamorphism in Ce based and La based metallic glasses^5^,^6^,^17^, is valuable to understand the polyamorphic transformation, which could be a general behavior in lanthanide based metallic glasses.

## Methods

To achieve Yb-based metallic glasses with good glass forming ability, constituent elements Zn, Mg and Cu were carefully selected and adjusted the composition. Metallic glass Yb_62.5_Zn_15_Mg_17.5_Cu_5_ ribbons were prepared using the single-roller melt-spinning technique.

### High-pressure PDF

Total synchrotron x-ray scattering data of the Yb_62.5_Zn_15_Mg_17.5_Cu_5_ metallic glass were collected at the sector 11-ID-B beamline at the Advanced Photon Source, Argonne National Laboratory with the incident beam size 150 μm × 150 μm and an 86.7 keV high energy. A 2D large amorphous-silicon-based flat-panel detector was used to record the synchrotron x-ray scattering patterns. The sample with width of 150 μm, length of 150 μm and thickness of 20 μm was located in the sample chamber which is T301 stainless steel gasket with 270 μm diameter hole between two anvils of diamond anvil cell (DAC). 1:4 methanol/ethanol and ruby are as pressure medium and marker, respectively. The pressure was up to 38.4 GPa and measured by the ruby fluorescence method with error bars of 0.1~0.2 GPa.

Raw image data were processed using software Fit-2D^27^ with masking strategy^28^ to remove the diamond peaks to obtain one-dimensional scattering data. Subtracting the contributions from the sample environment and background, the reduced pair distribution function *G(r)* and structure factor *S(Q)* were extracted using the program PDFgetX2^29^, which carries out a numerical Fourier transform of *S(Q)* according to


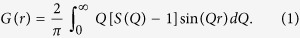


### High-pressure resistivity measurements

*In situ* electrical resistance was measured by a four-probe resistance system in a DAC. An aluminum oxide (Al_2_O_3_)/epoxy mixture powder layer was inserted between the T301 stainless steel gasket and diamond anvil to provide electrical insulation for the electrodes and gasket. Four electrodes made of platinum foil with a thickness of 10 μm were arranged to contact the sample in the chamber. The pressure was determined by a ruby and up to 40.9 GPa. The resistance was calculated from the Van de Pauw method^30^.

## Additional Information

**How to cite this article**: Li, L. *et al*. Polyamorphism in Yb-based metallic glass induced by pressure. *Sci. Rep.* **7**, 46762; doi: 10.1038/srep46762 (2017).

**Publisher's note:** Springer Nature remains neutral with regard to jurisdictional claims in published maps and institutional affiliations.
